# Arrhythmia Patterns in Patients on Ibrutinib

**DOI:** 10.3389/fcvm.2021.792310

**Published:** 2022-01-03

**Authors:** Muhammad Fazal, Ridhima Kapoor, Paul Cheng, Albert J. Rogers, Sanjiv M. Narayan, Paul Wang, Ronald M. Witteles, Alexander C. Perino, Tina Baykaner, June-Wha Rhee

**Affiliations:** ^1^Department of Medicine, Division of Cardiovascular Medicine, Stanford University, Palo Alto, CA, United States; ^2^Department of Medicine, Medical College of Wisconsin, Wauwatosa, WI, United States; ^3^Department of Medicine, Division of Cardiology, City of Hope National Cancer Center, Duarte, CA, United States

**Keywords:** cardio-oncology, tyrosine kinase inhibitor, atrial fibrillation, ventricular arrhythmia, ibrutinib, ambulatory event monitor

## Abstract

**Introduction:** Ibrutinib, a Bruton's tyrosine kinase inhibitor (TKI) used primarily in the treatment of hematologic malignancies, has been associated with increased incidence of atrial fibrillation (AF), with limited data on its association with other tachyarrhythmias. There are limited reports that comprehensively analyze atrial and ventricular arrhythmia (VA) burden in patients on ibrutinib. We hypothesized that long-term event monitors could reveal a high burden of atrial and VAs in patients on ibrutinib.

**Methods:** A retrospective data analysis at a single center using electronic medical records database search tools and individual chart review was conducted to identify consecutive patients who had event monitors while on ibrutinib therapy.

**Results:** Seventy-two patients were included in the analysis with a mean age of 76.9 ± 9.9 years and 13 patients (18%) had a diagnosis of AF prior to the ibrutinib therapy. During ibrutinib therapy, most common arrhythmias documented were non-AF supraventricular tachycardia (*n* = 32, 44.4%), AF (*n* = 32, 44%), and non-sustained ventricular tachycardia (*n* = 31, 43%). Thirteen (18%) patients had >1% premature atrial contraction burden; 16 (22.2%) patients had >1% premature ventricular contraction burden. In 25% of the patients, ibrutinib was held because of arrhythmias. Overall 8.3% of patients were started on antiarrhythmic drugs during ibrutinib therapy to manage these arrhythmias.

**Conclusions:** In this large dataset of ambulatory cardiac monitors on patients treated with ibrutinib, we report a high prevalence of atrial and VAs, with a high incidence of treatment interruption secondary to arrhythmias and related symptoms. Further research is warranted to optimize strategies to diagnose, monitor, and manage ibrutinib-related arrhythmias.

## Introduction

Atrial fibrillation (AF) is the most common sustained arrhythmia in the world, affecting at least 33 million individuals. The burden of AF has been rapidly increasing worldwide due to growing awareness and the broader application of portable event monitors and wearables and also due to shifts in demographics and an increase in the prevalence of risk factors (1). Moreover, with the growing use of cancer therapies in clinic, antineoplastic agents such as paclitaxel, mitoxantrone, doxorubicin, and TKIs have been associated with an increased risk of developing AF (2–5).

Ibrutinib is a Bruton's TKI that is used in a growing number of hematologic malignancies. It irreversibly binds Bruton's tyrosine kinase, which plays a critical role in B-cell development and proliferation, and thereby exerts its anticancer activity primarily in B-cell malignancies including chronic lymphocytic leukemia, mantle cell lymphoma, and Waldenström's macroglobulinemia (6). The use of ibrutinib has been associated with increased incidence of AF (5); with limited data on its association with other arrhythmias. These arrhythmias lead to a relatively high treatment interruption rate and cause significant morbidity in this patient population (4). There are limited data to date that comprehensively analyze both atrial and ventricular arrhythmia (VA) burden in patients on ibrutinib, and subsequent referral to subspecialty care, antiarrhythmic drug use, and treatment interruption patterns. Therefore, we hypothesized that long-term event monitors, as defined by continuous ECG monitoring >48 h, could reveal a high burden of atrial and VAs in patients on ibrutinib therapy which may lead to treatment cessation.

## Methods

We performed a single-center, retrospective cohort study to analyze consecutive patients on ibrutinib therapy, who had event monitors of at least 3 days of duration for any indication while on ibrutinib therapy between the years 2014 and 2021.

### Data Source and Covariates

Patient data including demographics, past medical history, history of AF, echocardiographic data (including left ventricular ejection fraction (LVEF), left atrial volume index (LAVI), and left atrial diameter), 12-lead ECGs, and event monitors with autotriggers were collected from electronic medical records. Event monitors were manually reviewed to confirm the diagnosis of AF, patterns of other arrhythmias seen, and assess the types of ventricular tachycardia (monomorphic vs. polymorphic). CHA_2_DS_2_-VASc score was automatically calculated from these data using age, sex, history of heart failure, hypertension, stroke, TIA, vascular disease, and diabetes.

### Outcomes

We compared the cohort that had AF seen on the event monitor against the cohort that did not, and the cohort that had ibrutinib held vs. those in whom ibrutinib was continued. We also conducted univariate analyses to identify the correlation between the development of AF and any clinical risk factors including ECG and echocardiographic parameters, and also a correlation between ibrutinib being held and any clinical risk factors.

#### Statistics

Statistical analyses were done using SPSS version 27 (IBM SPSS Statistics for Mac, IBM Corporation, Armonk, NY). Continuous data are reported as mean ± standard deviation, unless otherwise stated, and are tested for normality using the Shapiro–Wilk test (*p* > 0.05). Independent-samples *t*-test and Mann–Whitney U test were run to determine whether there were differences in mean values between cohorts and for analysis of continuous data. Categorical variables were compared using the Pearson's chi-squared test or Fisher's exact test where expected frequencies were <5. Statistical significance was assumed at the 5% level. This study was approved by the Institutional Review Board of Stanford University.

## Results

### Clinical Characteristics

**Figure 1:**
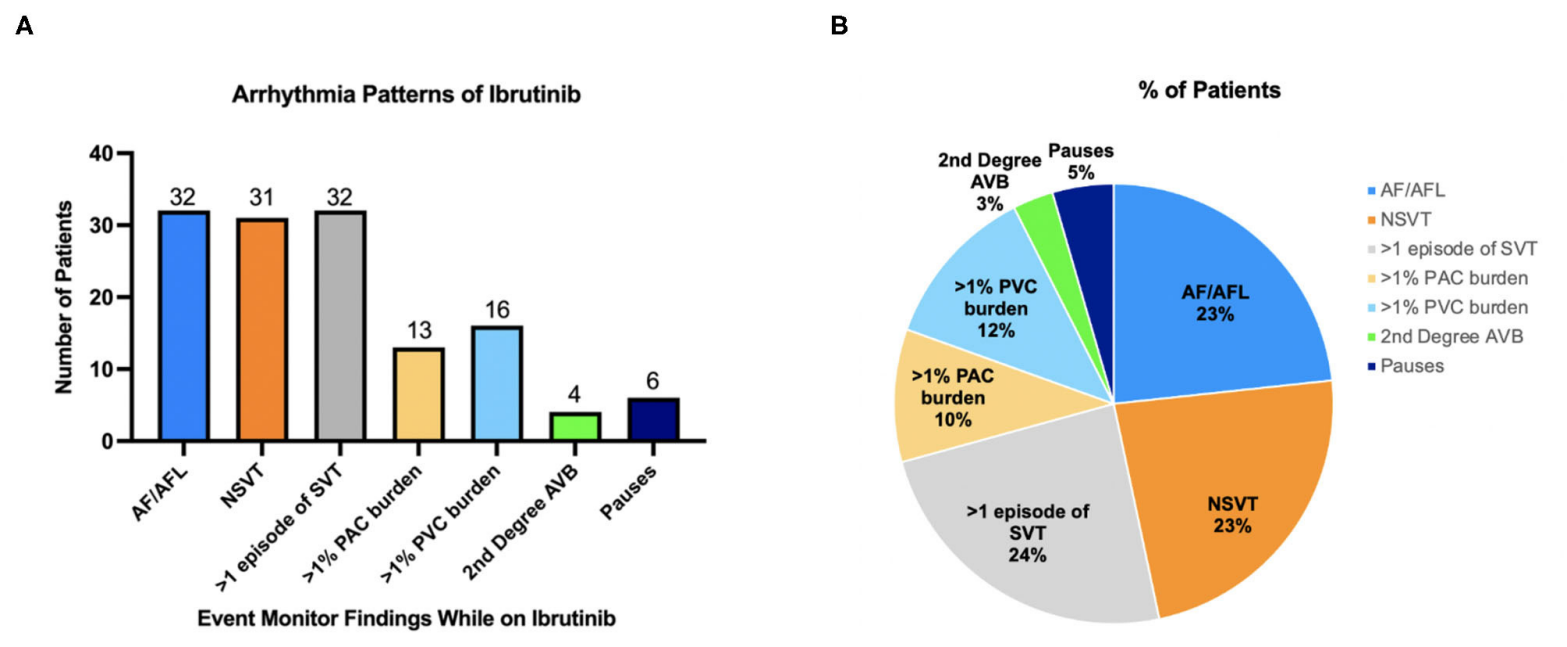
**(A)** Arrhythmias noted on cardiac monitor while on ibrutinib. **(B)** Distribution of arrhythmias during ibrutinib treatment. The pie chart depicts breakdown of total arrhythmia events detected and showing what the pattern of arrhythmias is on ibrutinib therapy as a percentage of all arrhythmias seen.

Of 755 patients who were on ibrutinib therapy for hematologic malignancies at Stanford Hospital between 2014 and 2019, 72 patients had event monitors (Zio, iRhythm Technologies, Inc., CA) while on ibrutinib therapy and were included in this analysis (Table 1). Thirteen patients (18%) carried a diagnosis of AF prior to ibrutinib therapy but the majority of the patients did not have a screening Holter monitoring, and therefore, the burden of pre-ibrutinib therapy arrhythmia is unknown. The most common indications for event monitoring included atrial arrhythmias (50%), palpitations (23%), abnormal EKG (14%), and syncope (6%). The 72 patients who were included in the analysis had a mean age of 76.9 ± 9.9 years, 25% were women, 68% with a diagnosis of hypertension, 62% with hyperlipidemia, 13% with COPD, 10% with prior history of cardiac surgery, mean BMI of 24.8 ± 4.1, and mean CHA_2_DS_2_-VASc score of 4 ± 2 (Figure 1A). The mean LVEF was 58.1 ± 9.1% and the mean LAVI was 36.4 ± 13.0 (ml/m^2^). Thirteen (18%) patients had a history of AF prior to initiation of ibrutinib. The average duration of time on ibrutinib therapy for all patients with event monitors was 31.9 ± 22.3 months. The median number of months on ibrutinib therapy was 28 months (range 1–111 months).

**Table 1:** Baseline demographics for patients undergoing ibrutinib therapy with cardiac monitor while on ibrutinib, divided by patients in whom therapy was held vs. continued.

**Characteristic**	**All patients (*n* = 72)**	**Patients in whom ibrutinib was held (*n* = 18)**	**Patients who continued ibrutinib (*n* = 54)**	***p***
Age (years)	76.9 ± 9.9	78.6 ± 11.2	76.3 ± 9.5	0.391
Sex (*N*, %)				0.753
Male	54 (75.0%)	13 (72.2%)	41 (75.9%)	
Female	18 (25.0%)	5 (27.8%)	13 (24.1%)	
Body mass index (kg/m^2^)	24.8 ± 4.1	24.2 ± 3.4	25.1 ± 4.3	0.520
LA volume index (ml/m^2^)	36.4 ± 13.0	43.6 ± 16.3	33.6 ± 10.5	**0.008**
EF (%)	58.1 ± 9.1	55.1 ± 11.1	59.2 ± 8.1	0.204
**Comorbid medical conditions (** ***N*** **, %)**
Congestive heart failure	26 (36.1%)	9 (50.0%)	17 (31.5%)	0.157
Valvular disease	31 (43.1%)	9 (50.0%)	22 (40.7%)	0.492
Hypertension	49 (68.1%)	13 (72.2%)	36 (66.7%)	0.662
Hyperlipidemia	44 (61.8%)	10 (55.6%)	34 (63.0%)	0.557
Diabetes mellitus	13 (18.1%)	2 (11.1%)	11 (20.4%)	0.376
Coronary artery disease	38 (38.9%)	5 (27.8%)	23 (42.6%)	0.264
Obstructive sleep apnea	23 (31.9%)	7 (38.9%)	16 (29.6%)	0.466
Chronic kidney disease	28 (38.9%)	9 (50%)	19 (35.2%)	0.264
History of AF (prior to ibrutinib therapy)	13 (18.1%)	4 (22.2%)	9 (16.7%)	0.725
Duration of Ibrutinib therapy (months)	31.6 ± 22.3	25.6 ± 20.2	33.6 ± 22.8	0.190
Patients on antiarrhythmic drug therapy (*N*, %)	9 (12.5)	5 (27.8%)	4 (7.4%)	**0.038**
Patients on antiarrhythmic drug therapy that was initiated after ibrutinib treatment (*N*, %)	6 (8.3)	4 (22.2%)	2 (3.7%)	**0.031**
**Care team involvement (** ***N*** **, %)**				
General cardiologist	50 (69.4)	16 (88.9%)	34 (63%)	**0.039**
Electrophysiologist	20 (27.8)	8 (44.4%)	12 (22.2%)	0.068

### Arrhythmia Patterns on Long-Term Event Monitors

**Figure 2:**
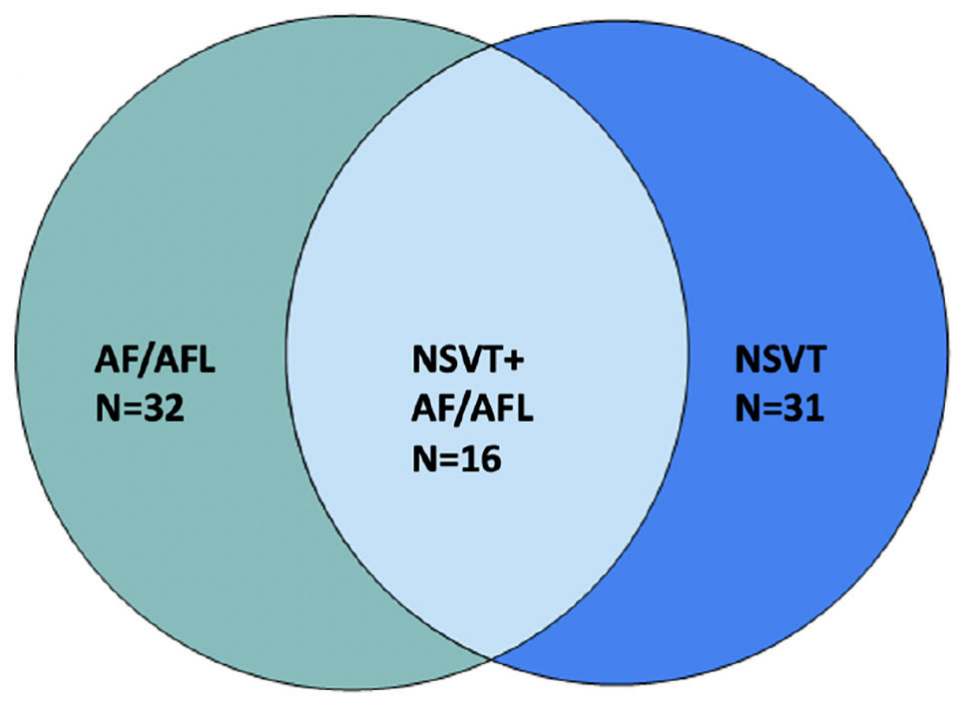
Overlap of atrial and VAs in patients on ibrutinib.

Most common arrhythmias documented were non-AF supraventricular tachycardia (SVT, in *n* = 32, 44.4% of patients), AF (*n* = 32, 44.4%), and non-sustained ventricular tachycardia (NSVT *n* = 31, 43.1%). Fourteen (19.4%) patients had >1% premature atrial contraction (PAC) burden; 16 (22.2%) patients had >1% premature ventricular contraction (PVC) burden (Figure 1B). Out of patients that had NSVT, five patients had polymorphic NSVT whereas the rest had monomorphic NSVT. Median QTc in patients with NSVT was 422 ms (range 375–507). Sixteen (22.2%) patients had both NSVT and AF recorded, which is about half of the population which had either NSVT or AF (Figure 2). A small proportion of these patients were followed by electrophysiologists (*n* = 20, 27.8%), whereas a higher proportion were followed by cardiologists (*n* = 50, 69.4%).

### Factors Associated With Ibrutinib Therapy Interruption

In 18 (25%) patients, ibrutinib therapy was held because of arrhythmias and/or related symptoms (Table 2). Six (8.3%) patients were started on antiarrhythmic drugs during ibrutinib therapy to manage these arrhythmias. Three patients required at least one direct current cardioversion (DCCV) for poorly controlled AF. Interruptions in ibrutinib therapy were associated with >1% PAC burden on event monitor while on ibrutinib therapy (*p* = 0.002) and a prior history of VT (*p* = 0.017); but not with the presence of the PVC burden of >1%, SVT, AF, or NSVT (all, *p* > 0.05) on the event monitor. Neither history of prior AF nor gender correlated with the frequency at which Ibrutinib was held. Patients in whom ibrutinib was held for arrhythmias were more likely to be seen by a cardiac specialist (*p* = 0.005), along with patients on ibrutinib whose Holter monitors showed NSVT (*p* < 0.001). Female patients were referred to a cardiac specialist less frequently than their male counterparts (*p* = 0.14).

**Table 2:** Detailed personalized information about patients in whom ibrutinib therapy was held.

**Patients in whom ibrutinib was held**	**Reason for ibrutinib interruption**	**Time on ibrutinib (months)**	**History of arrhythmia (AF or VT) prior to ibrutinib initiation**	**EF (%)**	**NSVT on Zio**	**QTc (ms)**	**Re-challenge**
Patient 1	New atrial flutter with rapid ventricular response	64	No	41	Yes	419	Yes
Patient 2	Symptomatic persistent AF	17	No	55	N/A	N/A	No
Patient 3	Persistent atrial flutter	23	AF	61	Yes	432	No
Patient 4	Recurrent AF	24	No	68	No	N/A	Yes
Patient 5	New symptomatic AF	5	No	57	No	N/A	No
Patient 6	AF, Tachyarrhythmia mediated LV dysfunction	31	No	42	Yes	445	No
Patient 7	Worsening of existing AF	18	AF	65	Yes	414	No
Patient 8	New AF, bleeding issues with anticoagulation	56	No	60	No	N/A	No
Patient 9	New AF	67	No	51	No	N/A	No
Patient 10	Symptomatic AF	22	No	69	No	N/A	No
Patient 11	Uncontrolled AF	13	AF	42	Yes	445	Yes
Patient 12	New AF	40	No	60	Yes	384	No
Patient 13	New AF	36	No	60	Yes	435	Yes
Patient 14	Symptomatic AF	3	No	70	Yes	410	No
Patient 15	New AF	1	No	57	No	N/A	No
Patient 16	New AF	15	No	56	No	N/A	Yes
Patient 17	New AF	23	No	35	Yes	486	Yes
Patient 18	Recurrent AF	4	AF	41	No	N/A	No

When looking at transthoracic echocardiography data, patients in whom ibrutinib was held for arrhythmia had a lower LVEF vs. those in whom ibrutinib was not held, albeit not statistically significant (55.1 ± 10.7 vs. 59.3 ± 8.2%; *p* = 0.09). However, for patients who had an LVEF ≤ 50%, 5 out of 12 (41.7%) had ibrutinib held for arrhythmias, which is considerably higher than the entire cohort (25%). Patients with a larger LA volume index had a higher probability of having ibrutinib held for arrhythmias (LAVI 43.3 ± 15.9 vs. 33.6 ± 10.7 ml/m^2^; *p* = 0.007). For those who were detected to have AF on event monitors (*n* = 32, 44%), EF was slightly lower (55.8 ± 8.9% vs. 60.0 ± 8.9%; *p* = 0.059), although it did not reach statistical significance.

There was no statistically significant relationship between AF on event monitor and risk factors such as age, hypertension, EKG, and echocardiographic parameters. No statistically significant difference was found between the cohort that developed AF and the cohort that did not. There was no statistically significant relationship between prior AF history and LA size or EF.

## Discussion

In this large dataset of long-term event monitors on patients treated with ibrutinib, we conduct detailed characterization of their arrhythmias which demonstrate a high burden of both atrial and VAs, with a high incidence of treatment interruption secondary to arrhythmias and a low rate of referral to specialists for arrhythmia management.

The incidence of atrial arrhythmias during ibrutinib therapy is well documented, ranging from 8 (7) to 14% (8) in prospective studies, and up to 40% in patients referred to cardio-oncology clinics (9). Compared with other TKIs, ibrutinib therapy has been the most consistent and independent risk factor associated with subsequent AF. These are several-fold higher than the reported incidence of both AF and NSVT on patients with non-cancer who received event monitors (10, 11). Despite the high incidence of AF in this population, it remains unknown which patients are at a higher risk for developing AF. While limited studies suggest advanced age, valvular disease, and prior history of AF to increase this risk (12, 13), these risk factors were not consistently found significant. Moreover, in this study, we did not find significant correlation with any clinical or demographic factors in patients who developed AF, which may be due in part to the limited sample size. We also did not find any significant correlation between the duration of the ibrutinib therapies and the development of AF. To better identify risk factors or predictors of ibrutinib-related AF, a more comprehensive large cohort study would be warranted.

In this study, ibrutinib therapy was held in 18 (25%) patients because of arrhythmias and/or related symptoms. We identified factors such as >1% PAC burden on event monitor while on ibrutinib therapy, a prior history of VT (*p* = 0.017), a high LA volume index, and low LVEF to be significantly associated with increased likelihood of ibrutinib therapy interruption due to arrhythmia or related symptoms. We believe a high LA volume index which correlates with high LA pressure and/or low LVEF may be significant as they can predispose the myocardium to develop subsequent arrhythmia. Otherwise, we were unable to obtain reliable data regarding rates of ibrutinib being held in the cohort that did not have event monitors. According to limited study reports available, rates of ibrutinib discontinuation are as high as 35% and AF seems to be the most common reason for ibrutinib being held in a comparable population of patients with hematologic malignancies (14, 15).

Data regarding VA during ibrutinib or other TKI therapies are rather scant. Some studies have used large registries of patients with cancer and looked at adverse events of VAs while on ibrutinib therapy. They found that even after accounting for baseline CV risk factors, ibrutinib was associated with a much higher incidence of VAs compared to similar patients not taking ibrutinib with a risk ratio up to 12.4 (16). When estimating the incidence of VAs in clinical trials involving ibrutinib, it was found that the incidence of VAs was significantly higher in patients receiving ibrutinib therapy compared to non-ibrutinib therapies (17). Yet, the detailed characterization, subtypes, and true incidence of VAs remain unknown as only symptomatic, clinical events were included in the analysis.

This study is unique in that it utilizes Holter event monitors which record all arrhythmic events, inclusive of both symptomatic and asymptomatic, over 2 weeks to comprehensively and unbiasedly characterize VAs among the patients treated with ibrutinib. In this study, the incidence of VAs was substantially higher with NSVTs captured in 43% of patients and a >1% burden of PVCs in up to 22% of symptomatic or arrhythmia-prone patients who were treated with ibrutinib and required Holter monitor screening. The observed rate of NSVT is an order of magnitude higher than the reported incidence of NSVT without known heart disease, which is generally in the range of 0.5–1% (18). Our results support the notion that ibrutinib is associated with a more frequent occurrence of VAs than previously believed. This finding also raises the question of underdiagnosis of VAs in patients treated with ibrutinib and emphasizes the need for further research in and more intensive monitoring of arrhythmias associated with ibrutinib therapy, and also other TKIs.

Multiple mechanisms have been proposed regarding the pathogenesis of TKI-induced arrhythmia. A recent study showed that off-target inhibition of C-terminal Src kinase (CSK), a non-receptor tyrosine kinase that inhibits Src kinase family members, may be responsible for the increased arrhythmogenicity seen with ibrutinib therapy (19). While CSK was reported to be expressed at a lower level in bulk ventricular vs. atrial tissue (19), it was found in both atrial and ventricular myocytes to a similar level (20) at the individual cell level which might explain the high burden of VAs observed in our study. Other proposed mechanisms for VAs due to ibrutinib include QTc prolongation and enhanced automaticity. In our cohort, the QTc of patients who developed NSVT was not found significantly prolonged (median duration of 422 ms).

Limitations of our study include patients enrolled in a single center, relatively small size of patients, and the absence of event monitors in all patients on ibrutinib. Notably, patients included in our study had an event monitor placed due to symptoms, ranging from palpitations to syncope, which can induce a selection bias to overestimate the incidence of arrhythmias in this patient population. Our cohort also consisted of older patients with a mean age of 77 years, more male patients, and patients with a modest burden of cardiovascular risk factors, all of which are known risk factors for developing atrial or VA. No statistically significant correlation was found between development of AF and clinical risk factors which have been shown to be related in larger studies such as age and hypertension, likely due to the small sample size of our cohort. Given the limited size of the cohort, only descriptive and univariate statistical analyses were performed. Additional clinical data such as alcohol intake data and prescription of other AF-inducing drugs could not be reliably obtained from our retrospective chart review and therefore not included in this analysis. Regarding non-AF SVT, we were unable to further classify the subtypes due to the limited quality of signals. Finally, we were unable to get the rates of ibrutinib discontinuation from the cohort that did not have event monitors placed to compare them to the patients included in this analysis. As such, a prospective and multicenter study would be warranted to better characterize arrhythmia associated with ibrutinib therapy.

## Conclusion

In this large dataset of Holter monitors on patients treated with ibrutinib, we find a significant burden of both atrial and VAs resulting in treatment interruption due to arrhythmias and related symptoms. Our results highlight the need for intentional monitoring and management of both atrial and VAs when patients are treated with ibrutinib therapy.

## Data Availability Statement

The raw data supporting the conclusions of this article will be made available by the authors, without undue reservation.

## Ethics Statement

This study was approved by the Institutional Review Board of Stanford University. Written informed consent for participation was not required for this study in accordance with the national legislation and the institutional requirements.

## Author Contributions

MF, RK, TB, and J-WR conceived and designed the study. MF and RK collected patient data. PC, AR, MF, RK, and TB analyzed the data. SN, PW, RW, and AP contributed to design the study and provided critical input on the manuscript. MF, RK, TB, and J-WR wrote the manuscript with input from all authors. All authors contributed to the article and approved the submitted version.

## Funding

This work is supported by National Institutes of Health Grants K08 HL148540 (J-WR), K08 HL153798 (PC), R01 HL83359 (SN), K23 HL145017 (TB), and American Heart Association Career Development Awards (J-WR and PC).

## Conflict of Interest

TB has received speaker and consultant fees from Biotronik, Medtronic, and PaceMate unrelated to this work. RW has received consulting fees from Pfizer, Alnylam, Eidos, and Ionis/Akcea unrelated to this work. AP has research support from the American Heart Association, Pfizer Inc. and Bristol Myers Squibb. Consultant for Abbott, Pfizer Inc. and Bristol Myers Squibb. The remaining authors declare that the research was conducted in the absence of any commercial or financial relationships that could be construed as a potential conflict of interest.

## Publisher's Note

All claims expressed in this article are solely those of the authors and do not necessarily represent those of their affiliated organizations, or those of the publisher, the editors and the reviewers. Any product that may be evaluated in this article, or claim that may be made by its manufacturer, is not guaranteed or endorsed by the publisher.
